# Impact of repeated co-treatment with escitalopram and aripiprazole on the schizophrenia-like behaviors and BDNF mRNA expression in the adult Sprague–Dawley rats exposed to glutathione deficit during early postnatal development of the brain

**DOI:** 10.1007/s43440-021-00318-z

**Published:** 2021-08-16

**Authors:** Marta A. Lech, Kinga Kamińska, Monika Leśkiewicz, Elżbieta Lorenc-Koci, Zofia Rogóż

**Affiliations:** 1 Department of Pharmacology, Maj Institute of Pharmacology, Polish Academy of Sciences, 12 Smętna Street, Kraków, Poland; 2 Department of Experimental Neuroendocrinology, Maj Institute of Pharmacology, Polish Academy of Sciences,, 12 Smętna Street, Kraków, Poland; 3 Department of Neuropsychopharmacology, Maj Institute of Pharmacology Polish Academy of Sciences, 12 Smętna Street, Kraków, Poland; 4 The Podhale State Higher Vocational School, Faculty of Cosmetology, Institute of Health, 71 Kokoszków, Nowy Targ, Poland

**Keywords:** Schizophrenia and glutathione deficit, Aripiprazole, Escitalopram, Social interaction test, Novel object recognition test, BDNF mRNA expression

## Abstract

**Background:**

Preclinical and clinical studies have indicated that impaired endogenous synthesis of glutathione during early postnatal development plays a significant role in the pathophysiology of schizophrenia. Moreover, some studies have suggested that antidepressants are able to increase the activity of atypical antipsychotics which may efficiently improve the treatment of negative and cognitive symptoms of schizophrenia.

**Methods:**

In the present study, we investigated the influence of repeated co-treatment with escitalopram and aripiprazole on the schizophrenia-like behavior and BDNF mRNA expression in adult rats exposed to glutathione deficit during early postnatal development. Male pups between the postnatal days p5–p16 were treated with the inhibitor of glutathione synthesis, BSO (L-buthionine-(S,R)-sulfoximine) and the dopamine uptake inhibitor, GBR 12,909 alone or in combination. Escitalopram and aripiprazole were given repeatedly for 21 days before the tests. On p90–92 rats were evaluated in the behavioral and biochemical tests.

**Results:**

BSO given alone and together with GBR 12,909 induced deficits in the studied behavioral tests and decreased the expression of BDNF mRNA. Repeated aripiprazole administration at a higher dose reversed these behavioral deficits. Co-treatment with aripiprazole and an ineffective dose of escitalopram also abolished the behavioral deficits in the studied tests.

**Conclusion:**

The obtained data indicated that the inhibition of glutathione synthesis in early postnatal development induced long-term deficits corresponding to schizophrenia-like behavior and decreased the BDNF mRNA expression in adult rats, and these behavioral deficits were reversed by repeated treatment with a higher dose of aripiprazole and also by co-treatment with aripiprazole and ineffective dose of escitalopram.

## Introduction

Schizophrenia is a chronic devastating psychiatric illness affecting about 0.5–1% of the world population [1, 2]. It develops progressively, remaining often undetected during childhood and adolescence, with the first episodes of psychosis that appear at early adulthood. The symptoms of the disorder can be divided into three main categories: positive symptoms, negative symptoms, and cognitive deficits [3]. Depression is a very important comorbidity occurring in approximately 50% of schizophrenic patients [4].

Schizophrenia is associated with neurodevelopmental, structural, and functional brain alterations, pathogenesis of which remains poorly understood. The prevailing hypothesis for the etiology of this disease assumes that both structural and functional abnormalities could be a consequence of multiple interactions between genetic and environmental factors during development [5] that set off a cascade of events extending into adulthood [6]. Although symptoms of schizophrenia are well characterized, a clear mechanism underlying the pathogenesis of this disease still remains unknown. However, oxidative stress as a consequence of the aberrant redox control has become an attractive hypothesis for explanation, at least partially, the pathophysiology of schizophrenia [7, 8].

Several studies have shown that the level of glutathione, the major antioxidant and redox regulator, is decreased in the cerebrospinal fluid and medial frontal cortex of drug-naïve schizophrenic patients [9] as well as in the post-mortem striatum [10] and prefrontal cortex of those treated earlier with antipsychotic drugs. In the periphery, significantly lower levels of glutathione were found in erythrocytes [11, 12] and plasma [10] in antipsychotic-free and chronically medicated schizophrenic patients in comparison to healthy control subjects.

The effects of glutathione deficit in the brain during development were studied in the animal models in adulthood [13–16]. Those studies demonstrated that chronic combined treatment of Osteogenic Disorder Shionogi (ODS) mutant rats, which, like humans, cannot synthesize ascorbic acid, with l-butionine-(*S,R*)-sulfoximine (BSO) and GBR12909 during early postnatal life induced schizophrenia-like memory deficits assessed in the novel object recognition test during adulthood. However, the effects of these compounds have not been studied in Sprague–Dawley rats, yet. The deficit in brain glutathione combined with DA reuptake inhibition during development caused a decrease in the number of dendritic spines of pyramidal neurons in the prefrontal cortex [17]. Therefore, morphological changes found in in vitro and in vivo studies could be related to morphological alteration reported earlier to occur in the prefrontal cortex of schizophrenic patients.

Glutathione, as the most abundant thiol antioxidant, plays a key role in the control of the redox state of cells and, thus in the regulation of various signaling pathways and gene expression. Hence, its deficiency can alter the functions of redox-sensitive receptor proteins and ion channels, such as NMDA and GABAA receptors, and calcium-activated potassium channels, which are involved in neurotransmission and synaptic plasticity. Changes in these redox-sensitive proteins may be associated with disturbances in the dopaminergic, glutamatergic, and GABA-ergic neurotransmitter systems known to be dysfunctional in schizophrenia [16, 18]. All these data seem to indicate that model substances, such as BSO and GBR 12,909, which, respectively, affect cell redox status and dopaminergic transmission, and may be useful for inducing the neurodevelopment rat model of schizophrenia.

Conventional, typical antipsychotics (i.e., antagonists of dopamine D_2_ receptors) commonly used in the treatment of schizophrenia mainly alleviate the positive symptoms [19, 20]. Contrary to typical antipsychotic drugs, atypical drugs partially alleviate the negative symptoms and slightly improve cognitive deficits [21, 22].

The pharmacology of aripiprazole is unique among atypical antipsychotics. It has a relatively high affinity for some monoaminergic receptors and acts as an antagonist of 5-HT_2A_ receptors as well as postsynaptic DA D_2_ receptors, and as a partial agonist of 5-HT_1A_ and presynaptic DA D_2_ receptors [23–27]. This drug partially relieves both positive and negative symptoms of schizophrenia.

Moreover, in several previous clinical and preclinical studies, it has been shown that the addition of antidepressants (ADs) to the therapy with atypical antipsychotics significantly increases their effectiveness in alleviating negative symptoms and improving cognitive tasks compared with the treatment with atypical antipsychotics alone [28–30]. The above data suggest that a new form of treatment of schizophrenia, combining ADs with atypical antipsychotics, may be of great importance in clinical practice. Furthermore, some earlier studies indicated a low level of the serum BDNF level in schizophrenic patients compared to control subjects [31, 32].

In light of the above data, the aim of our study was to evaluate the influence of repeated treatment with aripiprazole (an atypical antipsychotic drug) and AD escitalopram (ESC, a selective serotonin reuptake inhibitor) [33] given alone or in combination on the schizophrenia-like behavior and BDNF mRNA expression in adult rats exposed to glutathione deficit during early postnatal development.

## Materials and methods

### Animals and treatment

Pregnant Sprague–Dawley females at embryonic day 16 delivered by Charles River Company (Sulzfeld, Germany) were kept in individual cages under standard laboratory conditions; at room temperature of 21 ± 1 °C with 40–50% humidity on a 12-h light–dark cycle (the lights turned on at 7 a.m.), with free access to standard laboratory chow and tape water. One day after birth, the sex of pups was determined, and only males were left with their mothers to be used in further experimental procedure. Between the postnatal days p5 and p16, male Sprague–Dawley pups were treated with the selective inhibitor of glutathione synthesis, BSO (3.8 mmol/kg, *sc*, daily), and the inhibitor of dopamine reuptake GBR 12,909 (5 mg/kg, *sc*, every second day), alone or in combination. Control pups were given vehicle. On postnatal day, p23 rats were weaned and housed in groups of four until p92. ESC was given 30 min before aripiprazole repeatedly, once daily for 21 days before the tests. The last dose of the studied drugs was given 24 h before the test. Behavioral tests (social interaction and novel object recognition) evaluating expression of schizophrenia-like symptoms were carried out in adulthood (at p90-91 days of age). The tissue (hippocampus and prefrontal cortex) for biochemical assays was dissected on p92.

### Drugs and treatment

1-[2-[*Bis*-4(fluorophenyl)methoxy]ethyl]-4–3-(3-phenylpropyl)piperazine hydrochloride (GBR 12,909, Abcam Biochemicals, Cambridge, UK), L-butionine-(*S,R*)-sulfoximine (BSO, Sigma-Aldrich, Saint Louis, MO, USA), and escitalopram oxalate (ESC, Sigma-Aldrich, Saint Louis, MO, USA) were dissolved in 0.9% NaCl, while aripiprazole (Abcam Biochemicals, Cambridge, UK) was dissolved in 0.1 M tartaric acid. The pH of the solution was adjusted to 6–7 with 0.1 N NaOH. ESC (5 mg/kg, *ip*) and aripiprazole (0.1 and 0.3 mg/kg, *ip*) were administered once daily for 21 days using intraperitoneal (*ip*) injections in a volume of 2 ml/kg. All doses of drugs used in the present study were selected based on our earlier publications [34, 35].

### Compliance with ethical standards

All the experiments were performed in according with the EU directive 2010/63/EU and with the approval of the procedures by the Animal Care and Use Committee at the Maj Institute of Pharmacology, Polish Academy of Sciences, Kraków (permission on no 3/2018 of 11 January 2018). All efforts were made to minimize the number and suffering of animals used.

### Behavioral studies

#### Social interaction test

The social interaction test procedure was described previously by Górny et al. [35]. The behavior of the animals was measured over a 10-min period. The last dose of the studied drugs: ESC (5 mg/kg, *ip*) and aripiprazole (0.1 and 0.3 mg/kg, *ip*) was given 24 h before the test. Social interaction between two rats was expressed as the total time spent in social behavior, such as sniffing, genital investigation, chasing, and fighting with each other. The number of episodes was also counted. The social interaction test was performed on day p90. Each group consisted of 12 animals (six pairs).

#### Novel object recognition test

The procedure for the novel object recognition test was described previously by Górny et al. [35]. The novel object recognition test was performed 24 h after the last dose of drugs: ESC (5 mg/kg, *ip*) and aripiprazole (0.1 and 0.3 mg/kg, *ip*) administration. Next, the animals were placed in a box (T1 session) with two identical objects for 5 min. The time of object exploration was measured for each of the two objects separately. Then, 1 h after T1 session, the rats were placed again in the box (T2 recognition session) with two different objects: one the same as in the previous session (old) and another new for 5 min. The time of object exploration was measured for each of the two objects separately (sniffing, touching, or climbing). The novel object recognition test was performed on day p91. Each group consisted of eight to twelve rats.

##### BDNF mRNA expression analysis (real-time PCR)

The tissue (hippocampus and prefrontal cortex) for biochemical assays was dissected on p92. Freshly isolated rat tissues were stored at − 80 °C prior to the analysis. Total RNA was isolated using commercially available Bead-Beat Total RNA Mini Kit (A&A Biotechnology, PL) according to the manufacturer's instructions. After dissolving in water, RNA (1 μg) was reverse-transcribed to cDNA using High Capacity cDNA Reverse Transcription kit with RNAse inhibitor and random hexamers (MultiScribe™, Applied Biosystems, Life Technologies, Carlsbad, CA, USA). The BDNF mRNA level was determined by Real-Time PCR using predesigned TaqMan Gene Expression Assays (Applied Biosystems, UK). Assay IDs for the genes examined were as follows: BDNF (Rn01484925 m1) and for reference gene HPRT1 (Rn01527840 m1). Amplification was carried out in a total volume of 10 μl (FCx). The mixture containing: 1 × FastStart Universal Probe Master (Rox) mix (Roche, Germany), 900 nM TaqMan forward and reverse primers, and 250 nM of hydrolysis probe labeled with the fluorescent reporter dye FAM at the 5′-end and a quenching dye at the 3′-end and RNAse free water. We used 50 ng of cDNA for the PCR template, Real-Time PCR was conducted using thermal cycler Quant Studio 3 (Thermo Fisher Scientific, Waltham, MA, USA), and thermal cycling conditions were as follows: 2 min at 50 °C and 10 min at 95 °C followed by 40 cycles at 9 °C for 15 s and at 60 °C for 1 min. Each group consisted of eight rats.

### Statistical analysis

**Fig. 1:**
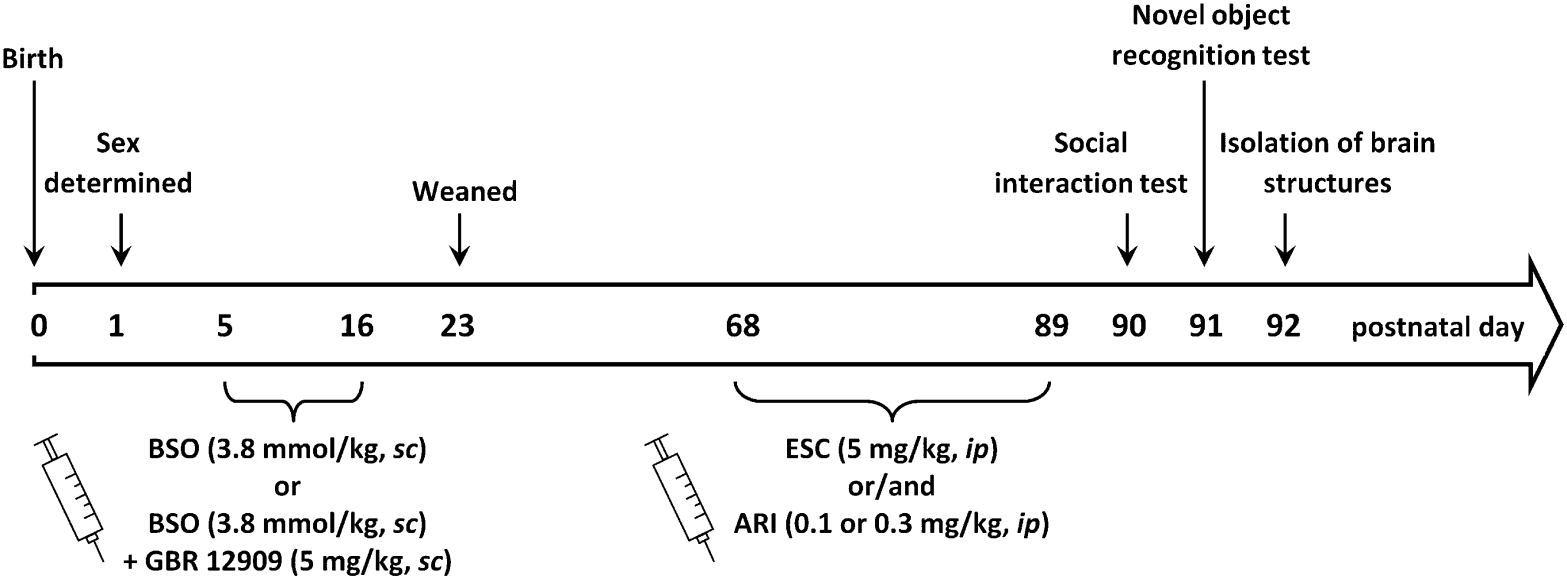
Timeline of the general protocol used in the present experiments. One day after birth, the sex of pups was determined, and only males were left with their mothers to be used in further experimental procedure. Between the postnatal days p5 and p16 male Sprague–Dawley pups were treated with the selective inhibitor of glutathione synthesis, BSO (3.8 mmol/kg, *sc*, daily), and the inhibitor of dopamine reuptake GBR 12,909 (5 mg/kg, *sc*, every second day), alone or in combination. Control pups were given saline. On postnatal day p23 rats were weaned and housed in groups of four until p92. ESC was given 30 min before aripiprazole repeatedly, once daily for 21 days before the tests. The last dose of the studied drugs was given 24 h before the test. Behavioral tests (social interaction and novel object recognition) were carried out in adulthood (at p90–91 days of age). The tissue (hippocampus and prefrontal cortex) for biochemical assays was dissected on p92

Statistical analysis of the obtained results was performed with the use the Statistica 64 v.13. Before the analysis, the data for each studied group were checked for normality using the Shapiro–Wilk test. The Levene’s test was used to check homogeneity of variance. Since all behavioral and biochemical data met both criteria, a one-way analysis of variance (ANOVA) for planed comparisons (the so-called contrast analysis) was applied. The recognition index was calculated for each rat [(time spent exploring the novel object—time spent exploring the familiar object)/(total time spent exploring both objects during the recognition session), and was expressed in percentages. The results are presented as the means ± SEM (standard errors of the means); they were considered statistically significant when* p* < 0.05 (Fig. 1).

## Results

Figure 1 presents the timeline of the general protocol used in the present experiments.

The effect of repeated co-treatment with escitalopram (ESC) and aripiprazole on the schizophrenia-like behaviors in the adult Sprague–Dawley rats exposed to glutathione deficit during early postnatal brain development

### The social interaction test in rats treated with BSO

**Fig. 2:**
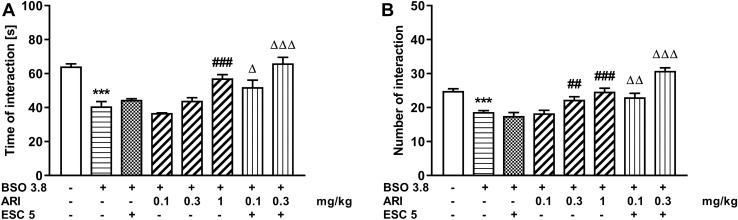
The effect of repeated co-treatment with escitalopram (ESC) and aripiprazole (ARI) on the deficits in the social interaction test performance in adult Sprague–Dawley rats induced by BSO (3.8 mmol/kg, *sc*) causing glutathione depletion during early postnatal brain development. The social interaction test performance in rats was assessed for 10 min in 90 days old rats by means of two parameters: the total time spent in social interaction (**A**) and the number of these interactions (**B**). ESC (5 mg/kg, *ip*) was given 30 min before administration of ARI (0.1 or 0.3 mg/kg, *ip*) once daily for 21 days. The results are shown as the mean ± SEM. Each group consisted of six pairs per group (12 rats). The statistically significant differences between the studied groups were calculated using a one-way ANOVA for planed comparisons. ***p < 0.001 *vs*. vehicle-treatment group; ^##^*p* < 0.01 and ^###^*p* < 0.001 *vs.* BSO-treatment group; ^Δ^*p* < 0.05, ^ΔΔ^*p* < 0.01 and ^ΔΔΔ^*p* < 0.001 *vs.* BSO + ESC-treatment group

The social interaction test was performed in 90-day-old rats that were chronically treated with the BSO model compound on the postnatal days p5-p16, and then in adulthood were chronically treated for 21 days with ESC (5 mg/kg) and aripiprazole (0.1, 0.3 and 1 mg/kg), alone or in combination. The analysis of the studied parameters, i.e., the time of interaction and the number of interactions, was performed using a one-way ANOVA for the planned comparisons (Fig. 2). This analysis performed for the interaction time (*F*_7,40_ = 20.33, *p* < 0.001) showed that administration of BSO in the early postnatal life significantly shortened the social interaction time assessed in adulthood, and chronic administration of 5 mg/kg ESC did not reverse this effect (Fig. 2A). Like ESC, aripiprazole at the tested doses of 0.1 and 0.3 mg/kg was ineffective in reversing the BSO-induced reduction in the social interaction time, but aripiprazole at 1 mg/kg was effective in reversing this effect. The combined treatment of an ineffective dose of ESC (5 mg/kg) with ineffective doses of aripiprazole (0.1 and 0.3 mg/kg) significantly (at the levels of *p* < 0.05 and *p* < 0.001, respectively) prolonged the time of social interaction compared to the BSO group receiving chronically ESC alone.

Similarly, a one-way ANOVA for planned comparisons (*F*_7,40_ = 33.83, *p* < 0.001) for the number of social interactions showed that BSO administered early in the postnatal life had a long-lasting effect, significantly reducing their number in adulthood (Fig. 2B). Chronic treatment with ESC (5 mg/kg) or aripiprazole at a dose of 0.1 mg/kg did not change the BSO-induced reduction in the number of social interactions between the two rats, but aripiprazole at doses of 0.3 and 1 mg/kg significantly increased their number. The combined treatment with an ineffective dose of ESC (5 mg/kg) and an ineffective dose of aripiprazole (0.1 mg/kg) significantly increased the number of social interactions between two rats compared to the BSO group treated with ESC alone (Fig. 2B). However, the effect of combined therapy on the number of social interactions was much greater when an ineffective dose of ESC (5 mg/kg) was administered with an effective dose of aripiprazole (0.3 mg/kg).

### The social interaction test in rats co-treated with BSO and GBR 12909

**Fig. 3:**
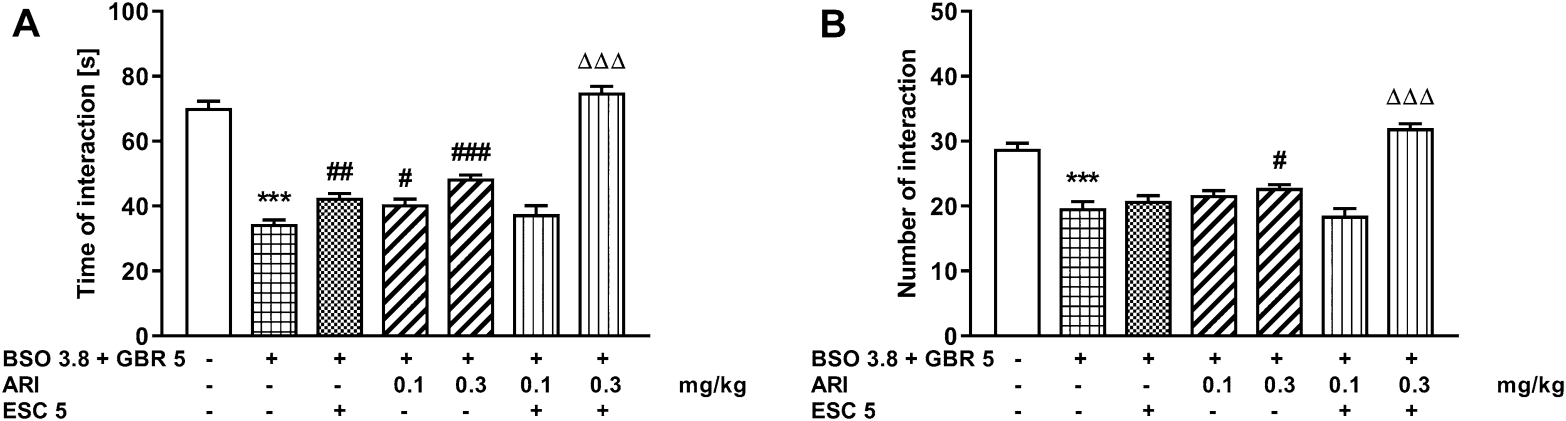
The effect of repeated co-treatment with escitalopram (ESC) and aripiprazole (ARI) on the deficits in the social interaction test performance in the adult Sprague–Dawley rats induced by BSO (3.8 mmol/kg, *sc*) given together with GBR 12,909 (5 mg/kg, *sc*) exposed to glutathione depletion during early postnatal brain development. The social interaction test performance in rats was assessed for 10 min in 91-day-old rats by means of two parameters: the total time spent in social interaction (**A**) and the number of these interactions (**B**). ESC (5 mg/kg, *sc*) was given 30 min before administration of ARI (0.1 and 0.3 mg/kg, *ip*), once daily for 21 days. The results are shown as the mean ± SEM. Each group consisted of six pairs per group (12 rats). The statistically significant differences between the studied groups were calculated using a one-way ANOVA for planed comparisons. *** *p* < 0.001 *vs*. vehicle-treatment group; ^#^*p* < 0.05. ^##^*p* < 0.01 and ^###^*p* < 0.001 *vs.* BSO + GBR 12,909-treatment group; ^ΔΔΔ^*p* < 0.001 *vs.* BSO + GBR 12,909 with ESC-treatment group

Similarly to the groups treated with BSO, also in the groups of rats receiving the combination of BSO + GBR 12909 model compounds in the early postnatal life, the social interaction test was performed in 90-day-old rats, and the studied parameters were analyzed by a one-way ANOVA for the planned comparisons (Fig. 3). The latter analysis carried out for the total time spent in social interactions (*F*_6,35_ = 84.08, *p* < 0.01) showed that, like in the case of BSO, also administration of the BSO + GBR 12909 combination in the early postnatal life resulted in a significant reduction in the time of social interactions (by 51%) assessed in adulthood (Fig. 3A). Chronic treatment with ESC (5 mg/kg) or aripiprazole (0.1 and 0.3 mg/kg) alone prolonged the social interaction time compared to the BSO + GBR 12909 group, and these effects although significant were only partial. Interestingly, administration of ESC (5 mg/kg) and aripiprazole at a dose of 0.1 mg/kg did not prolong the time spent in social interactions compared to the BSO + GBR 12909 group receiving chronically ESC alone. In contrast to the above effect, administration of ESC (5 mg/kg) and aripiprazole at a dose of 0.3 mg/kg significantly prolonged the social interaction time compared to the BSO + GBR 12909 group receiving chronically ESC alone (Fig. 3B). In the latter case, the time of social interactions was even slightly higher than in the control group (Fig. 3A).

As for the second studied parameter in the social interaction test, i.e., the number of social interactions, a one-way ANOVA for planned comparisons (*F*_6,35_ = 37.12, *p* < 0.001) showed a significant effect of the combined administration of model substances (BSO + GBR 12909) in early postnatal life on the number of social interactions assessed in adulthood (Fig. 3B). This analysis also showed that neither ESC (5 mg/kg) nor aripiprazole at a dose of 0.1 mg/kg administered chronically reversed the BSO + GBR 1290-induced decline in the number of social interactions, and only 0.3 mg/kg aripiprazole slightly but statistically significantly increased the number of these interactions (Fig. 3B). Also combined treatment with ineffective doses of ESC (5 mg/kg) and aripiprazole (0.1 mg/kg) did not increase the number of social interactions, but combined administration of 5 mg/kg ESC with effective dose of aripiprazole (0.3 mg/kg) significantly increased their number compared to the BSO + GBR 12909 group receiving chronically ESC alone (Fig. 3B).

### The novel object recognition test in rats treated with BSO

**Fig. 4:**
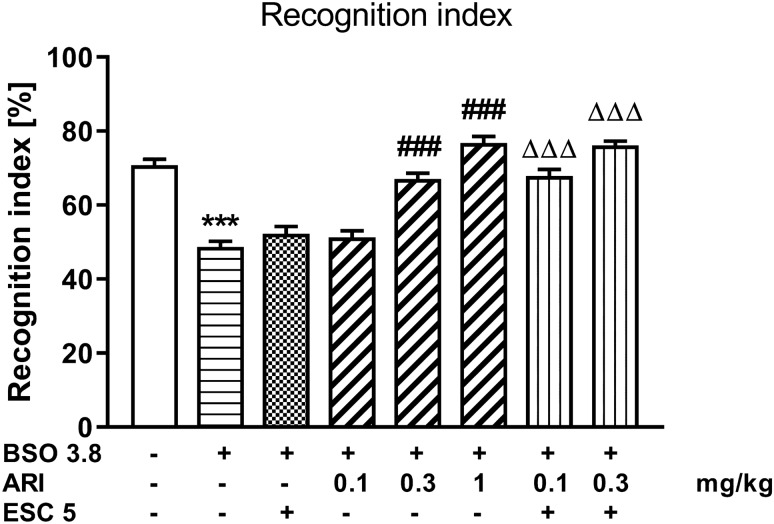
The effect of repeated co-treatment with escitalopram (ESC) and aripiprazole (ARI) on the deficits in the novel object recognition test performance in the adult 91 days old rats induced by BSO (3.8 mmol/kg, *sc*) causing glutathione depletion during early postnatal brain development. ESC (5 mg/kg, *ip*) was given 30 min before administration of ARI (0.1 and 0.3 mg/kg, *ip*), once daily for 21 days. Recognition index in the T2, recognition session was calculated for each rat [(time spent exploring the novel object—time spent exploring the familiar object)/(total time spent exploring both objects during the recognition session)], and was expressed in percentages. The results are shown as the mean ± SEM. Each group consisted of 12 rats. The statistically significant differences between the studied groups were also calculated using a one-way ANOVA for planed comparisons. ***p < 0.001 *vs.* vehicle-treatment group; ^###^*p* < 0.001 *vs.* BSO-treatment group; ^ΔΔΔ^*p* < 0.001 *vs.* BSO with ESC-treatment group

The novel object recognition test in rats treated with BSO was assessed at 91 days of age (Fig. 4). A one-way ANOVA for the planned comparisons performed for recognition indexes (*F*_7,88_ = 49.70, *p* < 0.001) calculated on the basis of the novel object recognition test showed that chronic BSO administration in the early postnatal days resulted in reduced memory retention in adult rats (Fig. 4). Chronic treatment with the higher doses of aripiprazole (0.3 and 1 mg/kg) abolished the BSO-induced memory deficits, while the lowest doses of aripiprazole (0.1 mg/kg) and ESC (5 mg/kg) were ineffective. Also, the combined administration of the ineffective dose ESC (5 mg/kg) with either the lowest dose of aripiprazole (0.1 mg/kg) or the effective dose of aripiprazole (0.3 mg/kg) significantly reversed cognitive deficits compared to the BSO group receiving chronic ESC alone (Fig. 4).

### The novel object recognition test in rats co-treated with BSO and GBR 12909

**Fig. 5:**
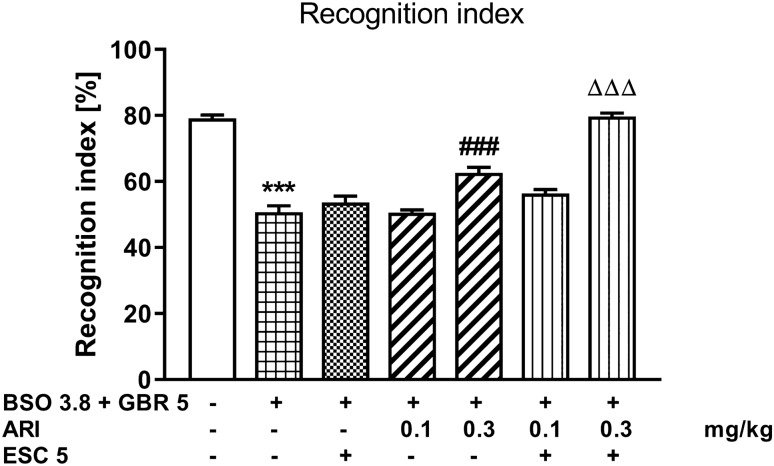
The effect of repeated co-treatment with escitalopram (ESC) and aripiprazole (ARI) on the deficits in the novel object recognition test performance in the adult 91-day-old rats induced by BSO (3.8 mmol/kg, *sc*) given together with GBR 12,909 (5 mg/kg, *sc*) exposed to glutathione depletion during early postnatal brain development. ESC (5 mg/kg, *ip*) was given 30 min before administration of ARI (0.1 and 0.3 mg/kg, *ip*), once daily for 21 days. Recognition index in the T2, recognition session was calculated for each rat [(time spent exploring the novel object—time spent exploring the familiar object)/(total time spent exploring both objects during the recognition session)], and was expressed in percentages. The results are shown as the mean ± SEM. Each group consisted of eight rats. The statistically significant differences between the studied groups were also calculated using a one-way ANOVA for planed comparisons. ***p < 0.001 *vs.* vehicle-treatment group; ^###^*p* < 0.001 *vs.* BSO + GBR 12,909-treatment group; ^ΔΔΔ^*p* < 0.001 *vs.* BSO + GBR 12,909 with ESC-treatment group

The novel object recognition test in rats co-treated with BSO and GBR12909 was assessed at 91 days of age (Fig. 5). A one-way ANOVA for the planned comparisons, performed for recognition indexes (*F*_6,49_ = 77.93, *p* < 0.001) and calculated on the basis of the novel object recognition test, showed that chronic administration the BSO + GBR 12909 combination in the early postnatal days led to disclosure of memory deficits in adulthood (Fig. 5). Chronic treatment with ESC (5 mg/kg) and a lower dose of aripiprazole (0.1 mg/kg) did not change memory deficits caused by the BSO + GBR 12909 combination, while a higher dose of aripiprazole (0.3 mg/kg) or combined chronic administration of ESC (5 mg/kg) and aripiprazole (0.3 mg/kg) reversed these memory deficits (Fig. 5).

The effect of repeated co-treatment with escitalopram (ESC) and aripiprazole on the BDNF mRNA expression in rats treated with BSO

### Frontal cortex

**Fig. 6:**
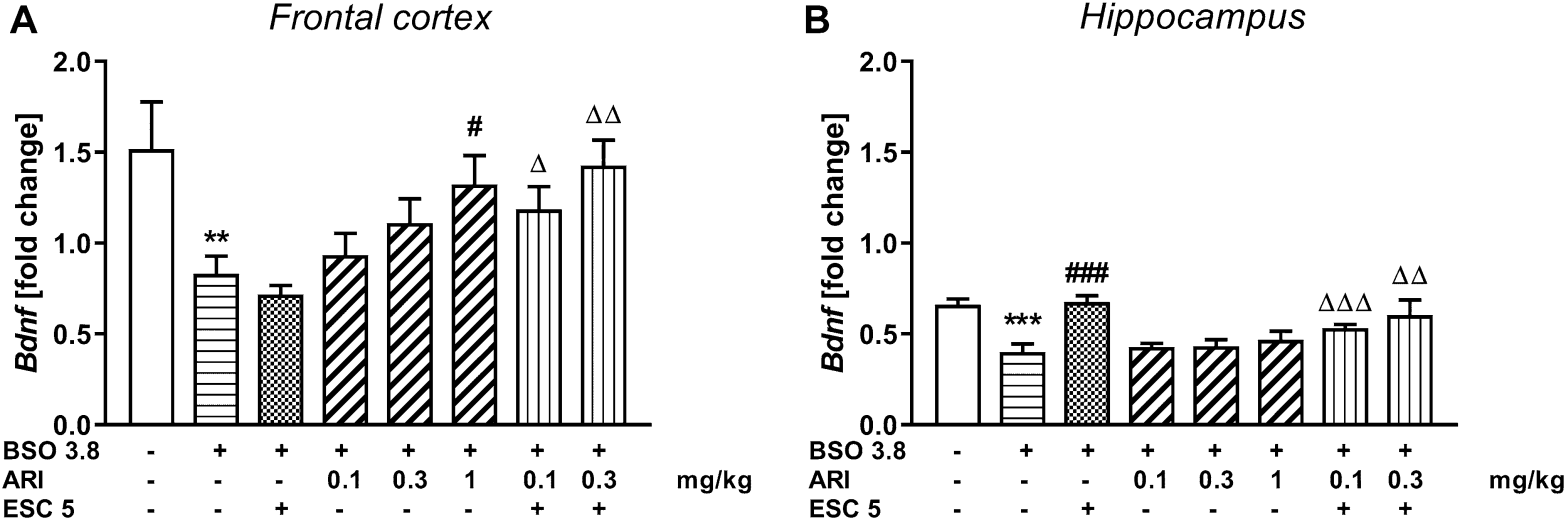
The effect of repeated co-treatment with escitalopram (ESC) and aripiprazole (ARI) on the BDNF mRNA expression in the frontal cortex (**A**) and hippocampus (**B**) in the adult 92-day-old rats in which glutathione deficit was induced by BSO administration (3.8 mmol/kg, *sc*) during early postnatal brain development. The results are shown as the mean ± SEM. Each group consisted of eight rats. The statistically significant differences between the studied groups were calculated using a one-way ANOVA for planed comparisons. **p < 0.01 and ***p < 0.001 *vs*. vehicle-treatment group; ^#^ *p* < 0.05 and ^###^*p* < 0.001 *vs.* BSO-treatment group; ^Δ^*p* < 0.05, ^ΔΔ^*p* < 0.01 and ^ΔΔΔ^*p* < 0.001 *vs.* BSO with ESC-treatment group

A one-way ANOVA for the planned comparisons performed for BDNF mRNA expression in the prefrontal cortex (*F*_7,56_ = 3.78, *p* < 0.002) showed that chronic BSO treatment in the early postnatal life significantly lowered the level of this parameter in adulthood, and chronic administration ESC (5 mg/kg) and lower doses of aripiprazole (0.1 and 0.3 mg/kg) did not reverse this effect, and only 1 mg/kg of aripiprazole was effective in reversing this change (Fig. 6A).

### Hippocampus

As in the prefrontal cortex, a one-way ANOVA for the planned comparisons performed for BDNF mRNA expression in the hippocampus (*F*_7,56_ = 4.545, *p* < 0.001) revealed that chronic BSO treatment in the early postnatal life significantly lowered the level of this parameter in adulthood (Fig. 6B). However, in the hippocampus, unlike the prefrontal cortex, chronically administered ESC reversed this effect, while the studied doses of aripiprazole were ineffective. In addition, the combined chronic administration of ESC (5 mg/kg) and 0.1 mg/kg of aripiprazole reduced the expression of BDNF mRNA compared to the BSO group chronically treated with ESC alone (Fig. 6B).

The effect of repeated co-treatment with escitalopram (ESC) and aripiprazole on the BDNF mRNA expression in rats treated with BSO and GBR 12909

### Frontal cortex

**Fig. 7:**
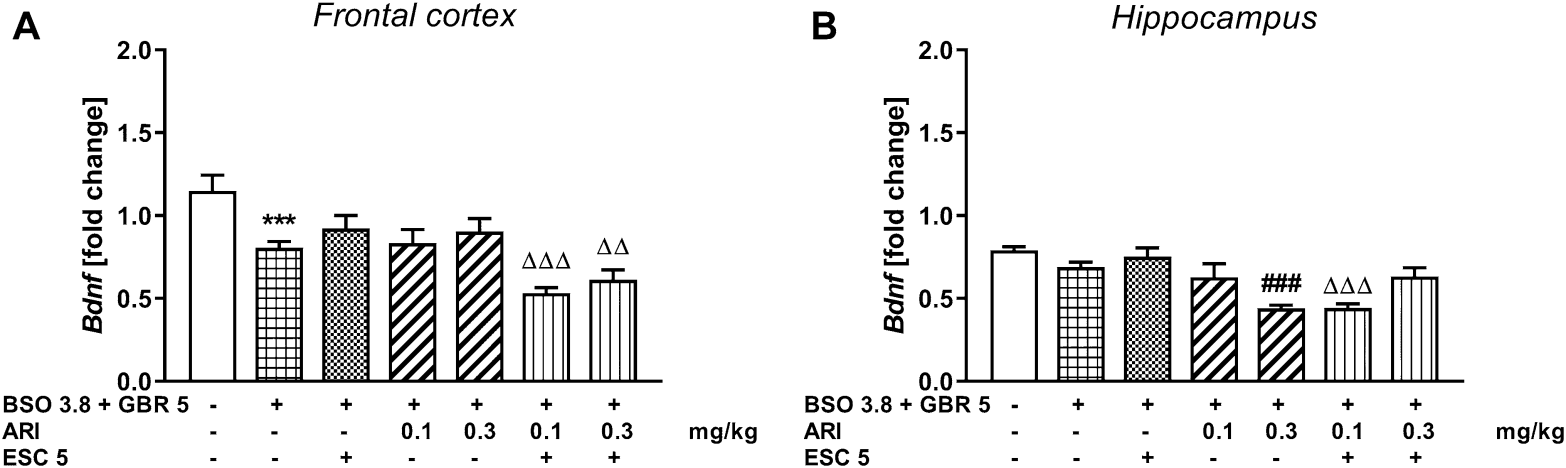
The effect of repeated co-treatment with escitalopram (ESC) and aripiprazole (ARI) on the BDNF mRNA expression in the frontal cortex (**A**) and hippocampus (**B**) in the adult 92-day-old rats exposed to glutathione deficit after BSO (3.8 mmol/kg, *sc*) given together with GBR12909 (5 mg/kg, *sc*) during early postnatal brain development. The results are shown as the mean ± SEM. Each group consisted of eight rats. The statistically significant differences between the studied groups were calculated using a one-way ANOVA for planed comparisons. ***p < 0.001 *vs.* vehicle-treatment group; ^###^*p* < 0.001 *vs.* BSO + GBR12909-treatment group; ^ΔΔ^*p* < 0.01 and ^ΔΔΔ^*p* < 0.001 *vs.* BSO + GBR 12,909 with ESC-treatment group

In the groups of rats receiving the combination of BSO + GBR 12909 model compounds in the early postnatal life, similarly to the groups treated with BSO, a one-way ANOVA for the planned comparisons for BDNF mRNA expression was performed in the prefrontal cortex (*F*_6,49_ = 8.653, *p* < 0.001) in adulthood (Fig. 7A). This analysis showed that such treatment significantly decreased expression of BDNF mRNA in this brain structure (Fig. 7A). However, neither ESC (5 mg/kg) nor aripiprazole at the doses tested did not reverse the BSO + GBR 12909-induced effect (Fig. 7A). Moreover, the combined chronic administration of ESC with both the lower and the higher dose of aripiprazole significantly decreased the levels of these parameters in the studied groups compared to the long-term administration effect of escitalopram alone (Fig. 7A).

### Hippocampus

A one-way ANOVA for the planned comparisons performed for BDNF mRNA expression in the hippocampus (*F*_6,49_ = 8.965, *p* < 0.001) showed that administration of the BSO + GBR 12909 combination in the early postnatal life did not induce changes in the expression of BDNF mRNA (Fig. 7B). Chronic treatment with ESC (5 mg/kg) or aripiprazole (0.1 mg/kg) alone had no effect on the expression of this parameter, while 0.3 mg/kg aripiprazole and the combined administration of ESC (5 mg/kg) + aripiprazole (0.1 mg/kg) decreased it significantly (Fig. 7B).

## Discussion

Our previous study [35] showed that inhibition of glutathione synthesis by repeated treatment with BSO alone or in combination with GBR 12909 to Sprague–Dawley pups in early postnatal life (p5-p16) induced the schizophrenia-like behavior evaluated in the social interaction test and in the novel object recognition test in early adulthood (p90). The above-mentioned behavioral tests are widely used to study some negative and cognitive symptoms of schizophrenia [36, 37]. In the present study, these behavioral deficits induced by treatment with model compounds (BSO or BSO + GBR 12 909) during early postnatal developmental were reversed by repeated treatment with a higher dose of aripiprazole as well as by co-treatment with ineffective doses of aripiprazole and ESC in adulthood. These chronic effects of the studied drugs, i.e., their ability to reverse the social and cognitive deficits of the schizophrenia type in the rat neurodevelopmental model of this disease, are consistent with acute effects of these drugs, used in the same dose range and combination, in previously described model of the MK-801-induced schizophrenia-like behavior [34, 38].

The behavioral data described above clearly indicated that the potentiation of the antipsychotic effect of aripiprazole by the used ADs in the MK-801-induced rat model of schizophrenic-type negative symptoms may be related to their action both through serotonin 5-HT_1A_ and dopamine D_1_ receptors [34]. In line with the latter study, it was also shown in the rat model of schizophrenic-type cognitive deficits induced by MK-801, the ADs potentiate the pro-cognitive effects of aripiprazole, and these effects may also be mediated by serotonin 5-HT_1A_ and dopamine D_1_ receptors [38]. In the above-cited study, in the conditions of combined administration of aripiprazole and ADs, an increase in extracellular concentrations of 5-HT and noradrenaline in the rat cortex was also shown, which may be of great importance to alleviating the negative symptoms and improving the cognitive functions [38]. Therefore, from the above study, it is reasonable to assume that the effects of combination treatment with aripiprazole and ESC on neurotransmitter release may be related to the activation or blockade of monoaminergic receptors in some structures of the rat brain, but not to pharmacokinetic interactions of these drugs, since clinical trials conducted in healthy subjects and patients with depression showed that aripiprazole did not significantly affect the pharmacokinetics of various classes of ADs (ESC, venlafaxine, fluoxetine, paroxetine, and sertraline) [39, 40].

Pharmacological models of schizophrenia induced by MK-801 or phencyclidine are symptomatic models used for screening antipsychotic drugs, and do not reflect the pathological changes leading to their formation. Looking for the biochemical markers of changes initiated in the early postnatal period by chronic administration of BSO alone or the combination of BSO + GBR 12909, in previous studies, we checked their impact on the levels of glutathione and sulfur amino acids (cysteine, methionine) and on the global DNA methylation in the prefrontal cortex and hippocampus [35]. These data suggest that transient alterations in the content of glutathione and sulfur amino acid methionine during early postnatal life lead to changes in epigenetic status in the prefrontal cortex and hippocampus, and to manifestation of social and cognitive deficits in adult rats. To further characterize this rat model of schizophrenia, in a recently published study [41], the activity of antioxidant enzymes (superoxide dismutase, catalase, glutathione peroxidase, and glutathione disulfide reductase) and the levels of lipid peroxidation in the prefrontal cortex and hippocampus of 16-day-old rats from the group treated with BSO + GBR12909 were analyzed in relation to glutathione content and sulfur amino acids, methionine, and cysteine [41]. This analysis showed that chronic administration of the BSO + GBR 12909 combination resulted in a significant reduction in the level of lipid peroxidation in the examined brain structures, indicating a weakening of the oxidative power of their cells and ultimately leading to changes in redox cell signaling. As a result of the redox state disturbance in the examined brain structures in the early postnatal life, social and cognitive deficits may occur in adulthood.

The brain-derived neurotrophic factor (BDNF) is the most abundant neurotrophin in the brain that, in addition to promoting neuronal survival and differentiation, modulates the efficacy of synaptic transmission [32, 42]. In animal models of depression and schizophrenia, BDNF levels were found to be abnormally regulated [32]. It has been shown that the level of BDNF is markedly reduced both in the plasma and post-mortem brains of patients suffering from schizophrenia, what suggests that BDNF dysfunction plays an important role in the pathogenesis of this disease [43]. Several in vivo studies have found that atypical antipsychotics increase the levels of both BDNF mRNA and its protein, while the typical antipsychotic drug haloperidol reduces or does not affect BDNF levels [43–46].

It is known that BDNF has an important role in neuronal development, synaptogenesis and also as a modulator of monoaminergic and GABA-ergic neurotransmitter systems [47, 48]. Thus, in our early experiments, we studied the long-term effects of chronic treatment with BSO and GBR 12909 alone or in combination in the early postnatal life on BDNF mRNA and its protein levels in the prefrontal cortex and hippocampus in adulthood in rats [49]. The obtained data showed that in the prefrontal cortex, BSO given alone or in combination with GBR 12909 induced a decrease in both BDNF mRNA and protein levels in adulthood. In the hippocampus, in rats treated only with BSO, also the decrease in the level of BDNF mRNA and its protein was observed. On the other hand, in the hippocampus of BSO + GBR-treated rats, no changes in the level of BDNF mRNA were observed, while there was a significant decrease in BDNF protein level when compared to the control group. In addition, treatment with GBR 12909 alone in early postnatal life modulates BDNF protein expression in adulthood in an opposite manner, reducing it in the prefrontal cortex and increasing it in the hippocampus. The above data suggest that in rats given GBR 12909 alone, this increase in the BDNF protein level in the hippocampus may be of compensatory nature, ultimately leading to the normalization of cognitive functions. However, the observed compensatory effect at the BDNF protein level did not occur in the rat hippocampus following co-treatment BSO with GBR 12909 [49]. Moreover, our and literature data suggest that redox imbalances [7, 18, 50, 51], as a result of repeated treatment with a glutathione synthesis inhibitor administered alone or in combination with a DA reuptake inhibitor in early postnatal development may be an important factor reducing BDNF protein expression in the prefrontal cortex and hippocampus in adulthood. In another model of schizophrenia [52], a decrease in the level of BDNF mRNA in the prefrontal cortex and the hippocampus was observed [53]. Also. other animal studies have shown that some early postnatal disturbances can have long-term effects on various neurotrophin-mediated processes, thereby affecting neuronal maturation and plasticity later in life [48, 54, 55].

Moreover, our present data indicated that the decrease in BDNF mRNA expression in the frontal cortex of rats treated with BSO was abolished by a higher dose of aripiprazole, and also by co-treatment with aripiprazole and ineffective doses of ESC. In contrast, in the hippocampus of rats treated with BSO + GBR 12909 and aripiprazole + ESC, even greater decrease in BDNF mRNA expression was observed than in rats treated with BSO and GBR12909. Our previous study showed that ESC (5 mg/kg, *ip*) administered repeatedly (once daily for 14 days) did not change in BDNF mRNA expression in the frontal cortex and hippocampus compared to the vehicle-treated group, but its higher dose (10 mg/kg, *ip*) increased this expression [56]. Moreover, it was demonstrated that repeated treatment with ESC (10 mg/kg) regulated intracellular pathway linked to neuroplasticity at both evaluated time points in an area-specific manner. For example, 7 days of treatment with ESC activated intracellular pathways linked to BDNF and increased the levels of pro-BDNF in the prefrontal cortex. On the other hand, 21 days of treatment with ESC decreased CREB/BDNF signaling, but increased p38 levels in the rat hippocampus. These data suggest that efficacy of ESC may be mediated by early and late effects on synaptic plasticity in selected brain areas [57]. In addition, it was demonstrated that aripiprazole reduced the total mRNA levels in the hippocampus (ventral and dorsal), while increasing it in the ventral hippocampus [46]. Our present experiments in which we used the whole hippocampus suggest that the decrease in the levels of the BDNF mRNA expression in rats treated with BSO + GBR 12909 and aripiprazole + ESC may be connected with this difference**.** Moreover, our present data suggest that redox imbalance after repeated treatment with a glutathione synthesis inhibitor alone or in combination with a DA reuptake inhibitor during early postnatal development may be an important factor which reduces the expression of BDNF protein in the prefrontal cortex and hippocampus in adulthood. Further studies are needed to investigate whether changes in the BDNF protein levels or mRNA expression in the prefrontal cortex and in the hippocampus of rats treated with BSO or BSO + GBR 12909 combination affect intracellular signaling pathways.

In conclusion, our present findings indicated that the inhibition of glutathione synthesis by repeated treatment with BSO alone and together with GBR 12909 in early postnatal development induced long-term deficits corresponding to schizophrenia-like behavior evaluated in the social interaction and novel object recognition tests, and decreased the expression of BDNF mRNA in the frontal cortex and hippocampus. Moreover, these behavioral deficits were reversed by repeated treatment with a higher dose of aripiprazole and also by co-treatment with ineffective doses of aripiprazole and ESC. The above data suggest that the neurodevelopment rat model of schizophrenia induced by glutathione deficit generated by repeated treatment with BSO alone and together with GBR12909 in early postnatal life may be very important for studies on the pathomechanism of schizophrenia.

## Abbreviations

Ads: Antidepressant drugs
ARI: Aripiprazole
BDNF: Brain-derived neurotrophic factor
BSO: l-butionine-(*S,R*)-sulfoximine
ESC: Escitalopram
GBR 12,909: 1-[2-[*Bis*-4(Fluorophenyl)methoxy]ethyl]-4–3-(3-phenylpropyl) piperazine
5-HT: Serotonin
GABA: Gamma aminobutyric acid
NMDA: *N*-Methyl-d-aspartate
